# Panacinar emphysema complicating idiopathic pulmonary hemosiderosis: a case report

**DOI:** 10.1093/omcr/omad091

**Published:** 2023-12-19

**Authors:** Talha Saleem, Mohammed J Al-Jaghbeer, Andrea V Arrossi Valeria, Atul C Mehta

**Affiliations:** Respiratory Institute, Cleveland Clinic Foundation, Cleveland, OH, USA; Respiratory Institute, Cleveland Clinic Foundation, Cleveland, OH, USA; Pathology and Laboratory Medicine Institute, Cleveland Clinic Foundation, Cleveland, OH, USA; Respiratory Institute, Cleveland Clinic Foundation, Cleveland, OH, USA

## Abstract

Idiopathic pulmonary hemosiderosis (IPH) is a rare entity with no known underlying etiology. It can be complicated by lung fibrosis. Emphysema is rarely reported as a consequence of IPH. We present a case of a 30-year-old female who presented with recurrent hemoptysis and shortness of breath. Radiographs revealed advanced emphysematous changes of the lower lobes. The diagnosis of IPH was established with an open lung biopsy. She was treated with systemic steroids, underwent bullectomy and was subsequently maintained on inhaled steroids.

## INTRODUCTION

Idiopathic pulmonary hemosiderosis (IPH) is a rare entity of unknown etiology [1]. Although primarily a childhood disease, the diagnosis can be made during adulthood. The clinical hallmark is recurrent episodes of diffuse alveolar hemorrhage (DAH) with no underlying etiology. The recurrence of hemoptysis eventually leads to hemosiderosis and fibrosis. To our knowledge, diffuse pan-acinar emphysema associated with IPH has been rarely reported. We present a case of a 30-year-old female, with remote history of smoking and an unusual combination of pulmonary hemosiderosis and pan-acinar emphysema.

## CASE REPORT

A 30-year-old female, an ex-smoker with a total smoking history of 1 pack year, having quit 2 years ago, presented with a 1-month history of dyspnea on exertion, fatigue and productive cough with intermittent hemoptysis. Amount of blood was approximately one eighth of a teaspoon, mixed with yellow phlegm. She denied any symptoms of gastroesophageal reflux, arthralgia, fever or loss of weight or appetite. There was no known history of marijuana, vaping or illicit drug use. She denied any exposure to asbestos, bird droppings or second-hand smoke. She denies any joint or vascular symptoms. There was no prior history of pneumothorax or any malignancy. There was no family history of emphysema, pneumothorax or Alpha-1-Antitrypsin (A1AT) deficiency.

**Figure 1:**
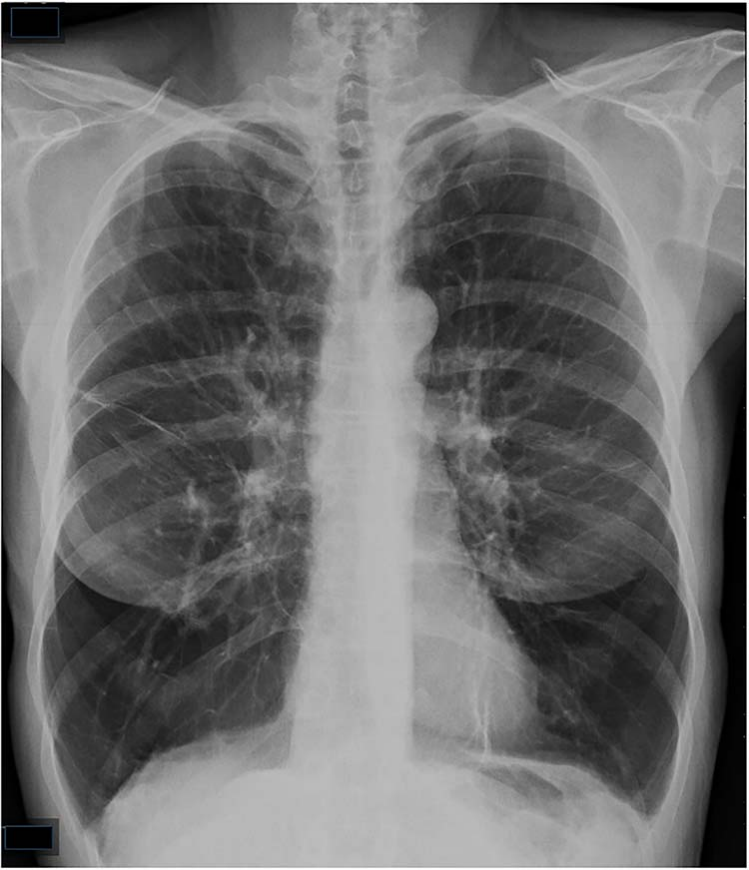
PA chest X-ray.

**Figure 2:**
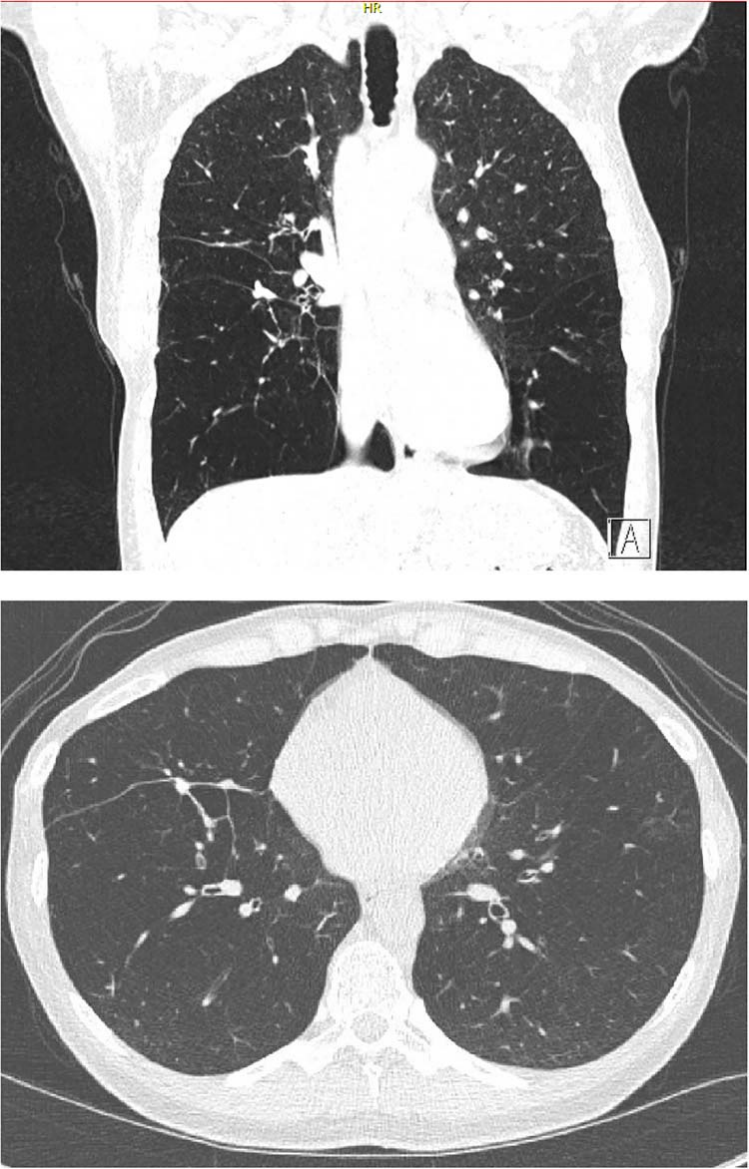
Coronal and axial sections of the chest CT.

Her physical examination was remarkable for bilateral expiratory wheezing and decreased breath sounds all throughout both lung fields. Skin examination showed a blistering diffuse maculopapular rash. A chest radiograph revealed bi-basilar hyperlucency (Fig. 1). She underwent a chest computed tomography (CT) scan that demonstrated hyperlucency of the lung parenchyma within the lower lung zones, compatible with panacinar emphysematous changes (Fig. 2). Additionally, there was a large bulla located within the right middle lobe.

Spirometry revealed moderate to severe obstructive ventilatory defect (FEV1/FVC- 57% predicted, FEV1–1.35 L [41% predicted]) with post-bronchodilator reversibility (post-bronchodilator FEV1 1.53 [47%]—13% change) with hyperinflation and air trapping (TLC 126% predicted and RV 3.28 L [188% predicted]). The diffusion capacity was reduced at 54% of but normalized for alveolar ventilation. Differential diagnosis included Emphysema due to A1AT deficiency, Parenchymal manifestation of connective tissue disease, and systemic vasculitis.

A serum A1AT level was normal with a PI*MM Genotype. Extensive workup, including liver functions, Human Immune Deficiency Virus and hepatitis panels were negative. A vasculitis panel, including anti-nuclear antibody, ESR and rheumatoid factor levels were normal. A panel of autoantibodies including anti-DNA Ab, anti-GBM Ab, anti-Jo-1 Ab, anti-PM-1 Ab, CK, anti-phospholipid antibody, anti-MPO and anti-PR3 was also negative. Complement levels were normal as well. She was found to have elevated IgE levels up to 195 kU/l (normal is <114 kU/L). Echocardiogram of the heart revealed a left ventricular ejection fraction of 65% with normal right ventricular function and trivial tricuspid regurgitation. She underwent a skin biopsy while being off all medications, which did not show any evidence of vasculitis. Her symptoms of hemoptysis failed to respond to a course of antibiotics.

**Figure 3:**
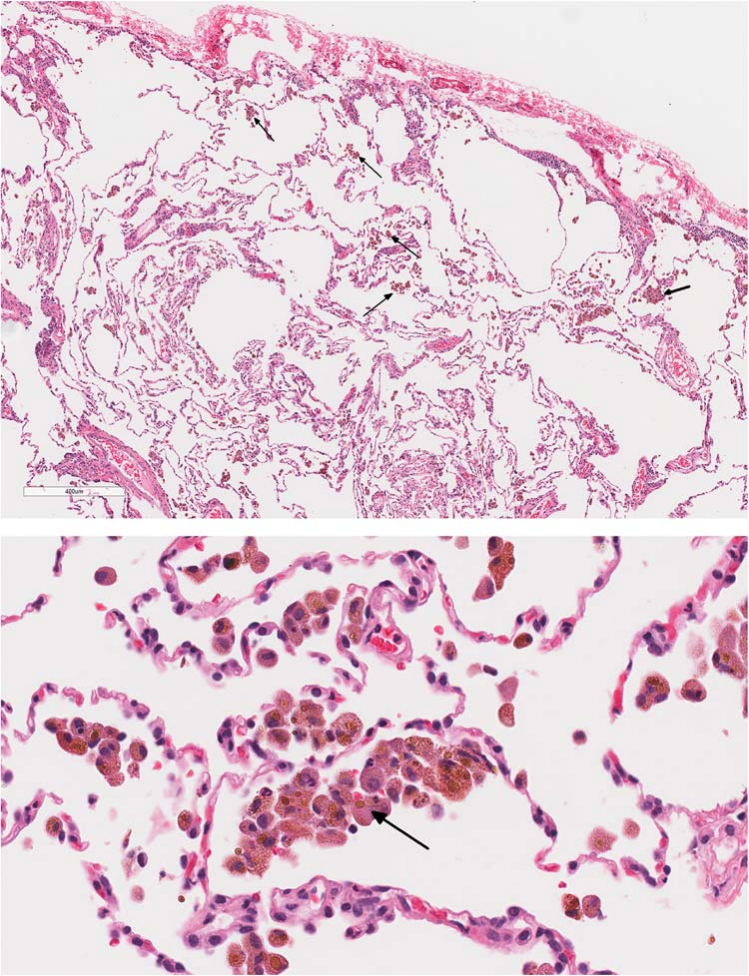
Emphysematous changes. Numerous alveolar hemosiderin-laden macrophages and calcium encrustation of the vascular walls.

Bronchoscopy with trans-bronchial biopsy failed to yield a diagnosis. Thus, a surgical lung biopsy was performed, which showed areas with numerous alveolar hemosiderin-laden macrophages and calcium encrustation of the vascular walls in keeping with prior episodes of alveolar hemorrhage, as well as foci of acute organizing alveolar hemorrhage. Additionally, Focal emphysematous changes were present. No vasculitis or capillaritis were present. Lymphangioleiomyomatosis was excluded by the lack of modified muscle cells (Fig. 3).

Given the lack of identifiable etiology for the pathologic findings, a diagnosis of IPH was established and the patient was started on treatment with intravenous methylprednisolone. The patient was evaluated by Dermatology for the skin rash and they believed it was a delayed reaction to Pulmicort Respules. The patient subsequently developed lip and tongue swelling, and dyspnea, deemed to be secondary to the methylprednisolone. Patient developed similar allergic reaction to Ipratropium bromide, Fluticasone propionate/Salmeterol (Advair), Montelukast and Fluticasone as well. She was treated with Fexofenadine with resolution of her symptoms. Medications were re-introduced to confirm reaction and allergy to Solumedrol was confirmed with a skin test. She was started on Dexamethasone weaning schedule. Patient continued to have episodes of hemoptysis and each episode improved with dexamethasone course for 3 weeks.

**Figure 4:**
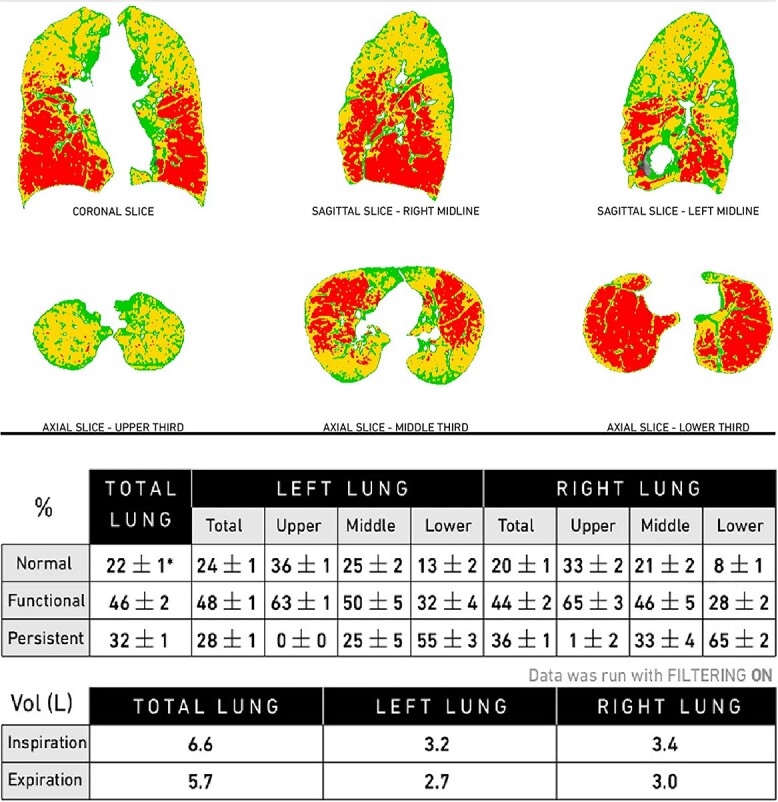
Quantitative chest CT scan—parametric response mapping.

The patient subsequently developed a post-surgical intercostal herniation of the lung, for which a right thoracotomy was performed for a bullectomy, right lung biopsy and repair of the chest wall hernia using prosthetic material. Pathology showed similar features to the prior sample, consisting of hemosiderosis and panacinar emphysema. Her symptoms were stabilized after the thoracotomy, and she was started on Formoterol Fumarate and Mometasone Furoate, the only inhalers she could tolerate. Since then, she has had poor yet stable lung function (Table 1). Repeat chest CT revealed stable emphysematous changes. We have continued to follow the patient for 15 years after the initial diagnosis, in which she he had no admissions to the hospital. Pulmonary functions (PFT) were mostly stable and a follow up quantitative chest CT (Fig. 4) showed clear lower lobe emphysema. The patient was deemed a poor candidate for a lung transplant secondary to the lack of social support.

**Table 1:** 

	Feb-03	Aug-05	Jun-06	Dec-18	Sep-10	Oct-12	Sep-14	Dec-17	Oct-19	May-20
FVC liters (% predicted)	3.17 (72)	3.05 (79)	3.21 (84)	2.26 (60)	2.66 (68)	2.88 (74)	2.47 (65)	2.22 (59)	1.54 (42)	2.33 (64)
FVC in liters Post-Bronchodilator (% Change)	3.2 (16)	3.13 (02)	3.2 (0)							
FEV1 in liters (% predicted)	1.35 (41)	1.17 (36)	1.07 (33)	0.79 (25)	0.97 (30)	0.97 (31)	0.84 (27)	0.75 (25)	0.56 (19)	0.71 (24.5)
FEV1 in liters Post-Bronchodilator (% Change)	1.53 (13)	1.40 (20)	1.10 (2)							
FEV1/FVC (%)	48.9 (57)	38.4 (41)	33.4 (40)	34.8 (42)	36.4 (44)	33.7 (41)	34.05 (42)	33.83 (42)	36.5 (45)	30 (37.5)
TLC in liters (% predicted)					7.36 (138)					7.3 (140.7)
RV in liters (% predicted)					4.24 (257)					4.55 (253.9)
DLCO ml/mmHg/min (% predicted)					24.66 (60)	24.4 (63)				10.64 (45.6)

## DISCUSSION

IPH is a rare disease of unknown etiology and prevalence [2]. Estimated incidence of 0.24 per million children per year was recorded from 1950 to 1979 in a Swedish study [3] as compared to 1.23 per million per year in a Japanese study [4]. Over 80% of cases occur among children, mostly in their first decade of life [5] with balanced sex distribution [6]. Remaining cases constitute adult-onset and/or undiagnosed childhood-onset IPH, mostly being diagnosed before the age of 30 years with inclination toward male predominance [7].

To date, the etiology remains unknown, yet a few hypotheses have been proposed. The presence of plasma circulating immune complexes and response to immune-suppressants therapy suggests an underlying auto-immune pathophysiology [8]. Additionally, celiac disease, [9–11] cow’s milk allergy [12, 13], exposure to insecticides [14] and fungi (especially Stachybotrys atra) [15], defects in iron metabolism [16], genetic pre-disposition [17] and a role of neutrophilic accumulation in alveoli [18], have also been linked to IPH.

Patients can present acutely with dyspnea, cough and hemoptysis. However, many have a more subtle presentation with worsening dyspnea, fatigue and anemia. Adults usually present with respiratory symptoms [19], while children tend to present with failure to thrive and anemia. Physical examination depends on the acuity of presentation. In an acute phase of intra-alveolar bleeding episode, there will be signs of respiratory failure. On the other end, the chronic phase of presentation, there will be pallor, failure to thrive and hepato-splenomegaly [2, 6, 19]. Rarely, no signs of disease are present on examination, as in our patient.

The complete blood count may reveal anemia, with an otherwise unremarkable workup like platelets count, coagulation profile, liver and kidney functions. Sputum examination can show erythrocytes and hemosiderin-laden macrophages pointing toward intra-alveolar bleeding. Broncho-alveolar lavage (BAL) has higher diagnostic yield than the sputum examination [20] exhibiting hemosiderin-laden macrophages, intact erythrocytes and occasional neutrophils [18].

PFTs in general show restrictive pattern [21] with reduced diffusion capacity (DLCO) in chronic cases and within normal limits when presenting acutely [22]. However, our patient PFTs demonstrated an obstructive pattern.

There is no pathognomonic imaging finding for IPH. During the acute phase, the chest radiographs show diffuse infiltrates, predominantly in the lower lung fields, with corresponding ground-glass attenuation on the CT scan [2, 23]. Subsequently, these shadows are absorbed with treatment with limited interstitial damage. However, during the chronic phase, lung structure gets modified and variable degree of fibrosis is seen [2, 24]. In our patient, CT scan revealed panacinar emphysematous changes with clear predilection to the lower lobes. We believe that the lack of fibrotic changes on CT maybe attributable to presenting in the acute phase.

The diagnosis of IPH relies on the clinical presentation with excluding other etiologies for DAH. A clinical picture of hemoptysis, iron deficiency anemia, sputum/BAL analysis, imaging studies and PFTs including basic spirometry and D_L,CO_ measurements help suspect the diagnosis. The laboratory investigation for ANA, anti-double stranded DNA, ANCA (both perinuclear and cytoplasmic variants), anti-GBM antibodies, anti-phospholipid antibodies and RF allow to rule out other caused of DAH [22].

Lung biopsy may be required to establish the diagnosis, which reveals hemosiderin-laden macrophages (siderophages) and an absence of focal or diffuse smooth muscle cell proliferations, vascular malformations, malignancy, pulmonary infarcts, capillaritis/vasculitis, granulomatous inflammation or infectious agents [23].

The differential diagnosis of emphysema is wide in such a case. A1AT Deficiency must be ruled out in young patients presenting with Emphysema. Ehlers Danlos can present with parenchymal changes and pneumothorax [25], our patient did not have a personal or family history of pneumothorax or joint disease.

Complication of IPH and emphysematous change is very rarely reported. Ikeda *et al*. suspected that repeated alveolar hemorrhage and hemosiderin deposition might lead to destruction of the lung structure i.e. alveolar elastic fibers [26]. Stainer *et al*. reported peri-bronchovascular emphysema in three patients who were initially diagnosed with IPH, however years later became ANCA positive. They noted known association between ANCA associated vasculitis and emphysema with pathogenesis related to inflammatory mechanisms with the ANCA antibodies, or with noxious insults like proteolytic enzymes and free oxygen radicals. [27]. Gocho *et al.* described peribronchial hemosiderosis induced strictures potentially causing air trapping and damage to peripheral bronchioles and alveoli eventually leading to centrilobular emphysema [28]. Our case is different from above mentioned cases in the severity of distribution of emphysema i.e. panacinar emphysema and even leading to formation of bullae. Noxious insults associated with recurrent alveolar hemorrhage and hemosiderin deposition leading to destruction of lung interstitium is a plausible explanation.

Since it is a rare disease, there are no controlled treatment trials. Management is based upon personal experiences and anecdotal evidence. One approach has been use of systemic glucocorticoids, mostly in the acute phase of IPH, with favorable response [6, 7]. Some authors recommend a starting dose of ≤1 mg/kg/day for a couple of months followed by a tapered dose. There is favorable response to chronic oral corticosteroids, with fewer IPH exacerbations [2, 29]. Immunosuppressant agents, including azathioprine, hydroxychloroquine, cyclophosphamide and methotrexate, have been tried as well with variable results [2, 30–33]. Lung transplantation have been performed [34, 35] with a risk of recurrence in the transplanted lungs. Our patient’s symptoms stabilized after bullectomy. This approach has been previously reported to improve the dyspnea in patients with emphysema [36] but not reported in patients with IPH. Our patient developed a post-surgical thoracic wall herniation, which was reported in to occur spontaneously in a patient with IPH [37].

The lack of prospective data on patients with IPH makes prognostication difficult. The most frequent cause of death is massive DAH resulting in acute respiratory failure [2, 6]. Our case exhibited unusual features different from descriptions in prior studies of in terms of imaging and pathologic findings.
